# Diffuse alveolar hemorrhage in Wegener’s granulomatosis

**DOI:** 10.4103/0970-2113.76302

**Published:** 2011

**Authors:** Vineet Mahajan, Jagdeep Whig, Anil Kashyap, Sushil Gupta

**Affiliations:** *Department of Pulmonary and Critical Care, Dayanand Medical College and Hospital, Ludhiana, Punjab, India*; 1 *Department of Critical Care, Dayanand Medical College and Hospital, Ludhiana, Punjab, India*

**Keywords:** Alveolar hemorrhage, Wegener’s granulomatosis

## Abstract

Diffuse alveolar hemorrhage is a life-threatening though rare manifestation of Wegener’s granulomatosis (WG). An active diagnostic workup, intensive observation, and aggressive immunosuppressive treatment are cornerstones of the management. The treatment modalities available for such complications are pulse cyclophosphamide therapy with steroids. We report here a case of WG with diffuse alveolar hemorrhage as the first manifestation of the disease in life that responded to steroids and cyclophosphamide.

## INTRODUCTION

Wegener’s granulomatosis (WG) consists of necrotizing granulomatous disorder in the nose and lung, necrotizing granulomatous vasculitis in generalized arterioles, and intractable vasculitis that features renal necrotic crescent forming nephritis. It is the most common of the antineutrophil cytoplasmic antibody – associated vasculitis which also includes Churg–Strauss syndrome and microscopic polyangiitis.[1] Diffuse alveolar hemorrhage is a serious manifestation of WG with most studies showing a mortality of 60%, six times greater than vasculitis without pulmonary hemorrhage.[2] The diagnosis is difficult since the occurrence is abrupt and both symptoms and histology of the lesion are nonuniform and nonspecific. Since the early diagnosis is essential for the outcome, it should be considered in every patient of WG with severe pulmonary symptoms. Cyclophosphamide and corticosteroids are the recommended form of therapy for this disorder.

## CASE REPORT

A 35-year-old male was admitted to our department with complaints of nonproductive cough, dyspnea, hemoptysis, and low-grade fever for last 20 days. Dyspnea was slowly progressive with four to five episodes of hemoptysis. He was also complaining of muscle pain and lethargy along with rash over bilateral lower limbs. There was no past history of any allergic diathesis or bronchial asthma. He was a driver by occupation, nonsmoker, and occasional alcoholic.

**Figure 1:**
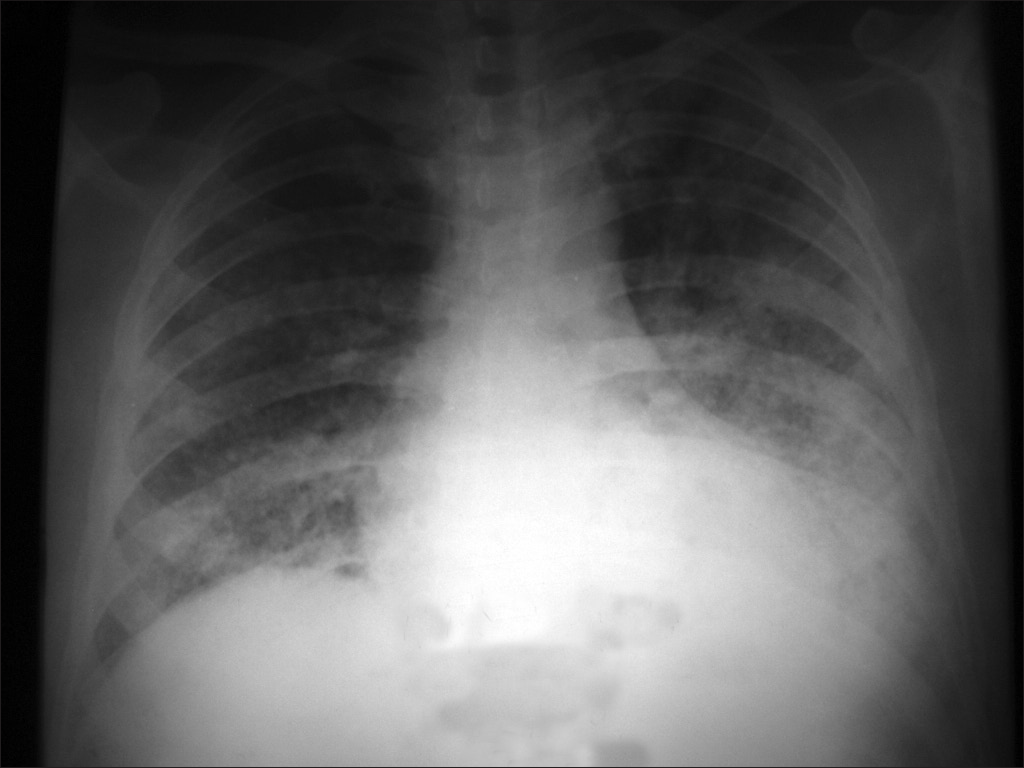
Chest radiograph showing bilateral diffuse alveolar infiltrates

**Figure 2:**
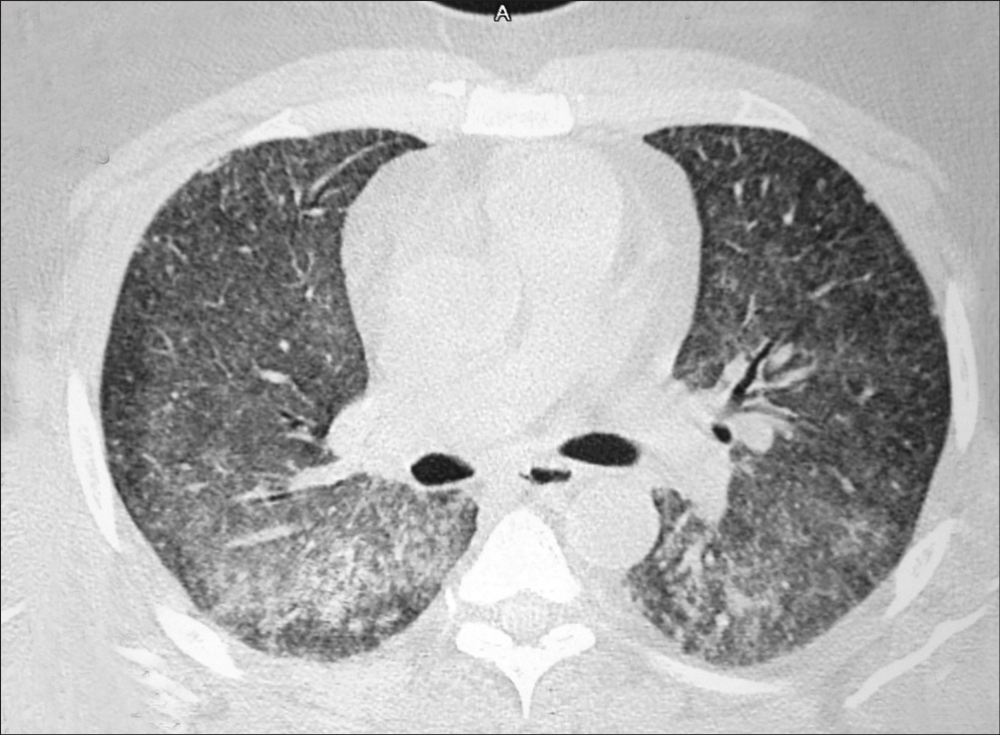
CT thorax showing diffuse ground glass opacities

On general examination, he was pale. His resting pulse rate was 128/min, blood pressure 100/70 mmHg, and respiratory rate 36/min. Chest examination was unremarkable on inspection, palpation, and percussion. On auscultation, bilateral diffuse course crepitations were audible. Routine investigations showed were as follows: Hb – 6.6 g%, TLC – 8600/mm^3^, DLC – neutrophils – 84%, lymphocytes – 13%, monocytes – 3%, platelet count – 592,000, erythrocyte sedimentation rate – 140, serum bilirubin – 0.4 mg/dl, SGPT – 48 IU and SAP – 142 IU, blood urea – 56 mg/dl, and serum creatinine – 1.2 mg/dl. Arterial blood gas analysis done at the time of admission revealed hypoxia with PaO_2_ 38 mmHg and PaCO_2_ 32 mmHg. Chest x-ray showed bilateral alveolar infiltrates [Figure 1]. High-resolution computed tomogram of chest revealed bilateral diffuse ground glassing [Figure 2].

**Figure 3:**
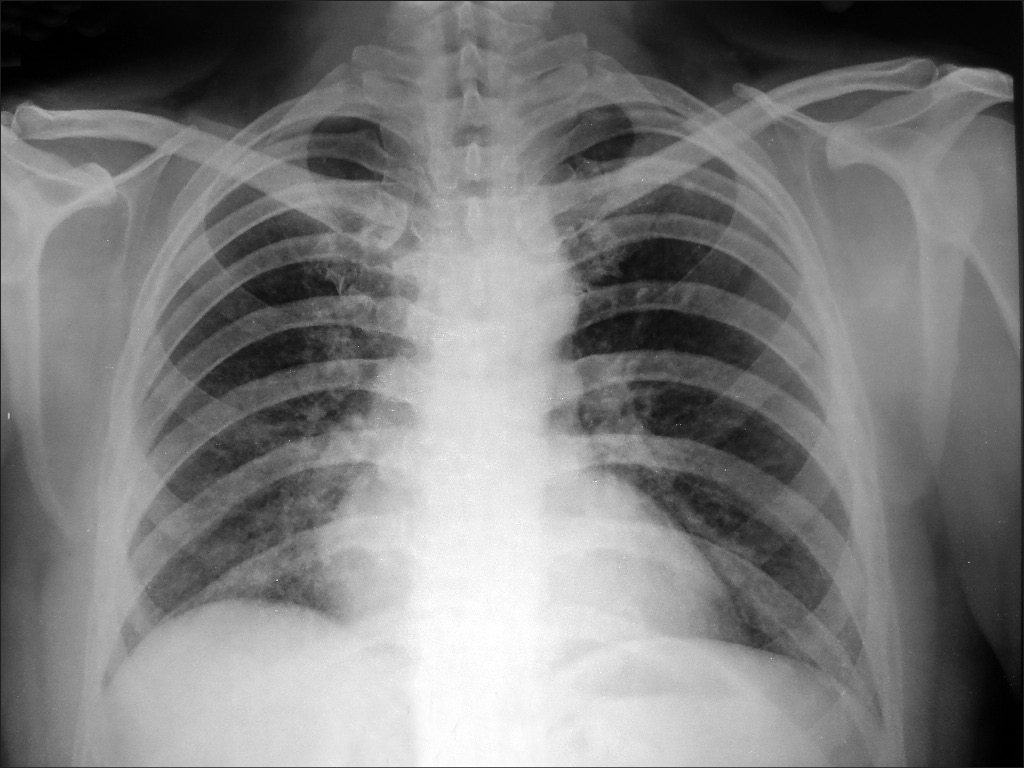
Follow-up chest radiograph showing significant radiological clearing

The triad of anemia, hemoptysis, and diffuse air-space consolidation prompted us to think of some systemic process and the patient was further investigated to establish the etiology. Urine microscopy revealed microscopic hematuria. Sputum samples for acid-fast bacilli were found to be negative on three consecutive days. The Mantoux test showed no induration after 72 h. Bleeding time and clotting time were within normal limits. Serology for dengue and leptospira antigens came out to be negative. Skin biopsy was consistent with the histology of leukocytoclastic vasculitis. Fundus examination revealed multiple flame-shaped superficial and deep hemorrhages consistent with vasculitis. ENT evaluation showed thick mucoid secretions with nasal biopsy showing multiple granulomas. Bronchoscopy was done and three consecutive Bronchoalveolar lavage (BAL) samples came out to be hemorrhagic consistent with alveolar hemorrhage. Later on BAL came out to be positive for hemosiderin-laden macrophages (>20% of total alveolar macrophages) strengthening our diagnosis. To rule out any systemic vasculitis, the antinuclear antibody (ANA) profile and antineutrophil cytoplasmic antibody (ANCA) were sent. The ANA profile was negative but c-ANCA came out to be strongly positive. So the diagnosis of diffuse alveolar hemorrhage was made and WG was thought to be the cause. The patient was given cyclophosphamide pulse (1 g) along with pulse methylprednisolone (750 mg) daily. Four units of blood were transfused to correct anemia and to improve the general condition. Noninvasive ventilation was used to maintain oxygenation. After about 48 h of strict monitoring, he showed significant improvement clinically and arterial blood gas with PaO_2_ 58 mmHg. Radiograph taken after 5 days of treatment showed significant clearing [Figure 3]. The patient is clinically asymptomatic after five cycles of cyclophosphamide pulse therapy.

## DISCUSSION

Diffuse alveolar hemorrhage (DAH) denotes diffuse intra-alveolar bleeding from small vessels as a result of severe damage of the alveolocapillary membrane of the lungs.[3] DAH may be a manifestation of systemic diseases, as well as a result of an injury restricted to the lungs. Most cases of DAH are caused by capillaritis associated with systemic autoimmune diseases such as antineutrophil cytoplasmic antibody-associated vasculitis, antiglomerular basement membrane disease, and systemic lupus erythematosus.[4] Rarely, pulmonary metastasis of angiosarcoma has also been reported as the cause of DAH.[5] Generally speaking, dyspnea, cough, hemoptysis, and new alveolar infiltrates in conjunction with bloody BAL specimens (with numerous erythrocytes and siderophages) establish the diagnosis of diffuse alveolar hemorrhage. Surgical biopsy from the lung or another organ involved by an underlying condition is often necessary.[6] However, up to one-third of patients do not have hemoptysis;[7] the alveolar infiltrates can be unilateral, and a drop in hematocrit or hemoglobin can be difficult to document. DAH can be diagnosed with BAL showing persistent or even increasing blood on three sequential aliquots from a single affected area of the lung.[6] In subacute or recurrent episodes, counting the hemosiderin-laden macrophages (siderophages) as demonstrated by Prussian blue staining of a pooled lavage specimen centrifugate may be useful for diagnosis as ≥ 20% siderophages out of total alveolar macrophages denotes DAH.[8] The diagnosis can also be suggested by an increase in the diffusion capacity of carbon monoxide of more than 30% over baseline.[9]

WG is a multisystem disease characterized pathologically by necrotizing granulomatous inflammation of the upper and lower respiratory tracts and glomerulonephritis. It is an uncommon disease with an estimated prevalence of 3 per 100,000, and the mean age of onset is 40 years.[10] The lung is the most commonly affected organ in WG with evidence of involvement in over 90% of patients during the course of their disease; in 9% it is the only organ affected.[7] It can manifest as asymptomatic infiltrates or may be clinically expressed as cough, hemoptysis, dyspnea, and chest discomfort. Although the lung is the most common organ system affected in WG, diffuse alveolar hemorrhage as a result of capillaritis is uncommonly seen with an estimated incidence of 7–45%.[11]

Typical findings on plain radiograph include bilateral, multiple rounded opacities ranging from a few millimeters to 10 cm in diameter. There is commonly cavitation of these nodules. Computed tomography may demonstrate nodules that are not apparent on radiography and is superior in demonstrating the presence of cavitation. Acute air space consolidation or ground glass opacities secondary to pulmonary hemorrhage is the second most common radiographic finding and may occur with or without the presence of nodules.[12] Nodules or masses occured in 89% and ground glass attenuation in 26% of 57 patients when CT findings were analyzed by Lohrmann *et al*.[13] The diagnosis of ANCA-associated vasculitis is made on the basis of the clinical findings, by biopsy of a relevant involved organ and the presence of ANCA. Testing for ANCA using both indirect immunofluorescence and antigen-specific enzyme-linked immunosorbent assay is recommended, and provides high sensitivity (approximately 99%) and good specificity (approximately 70%) in those with generalized Wegener’s.[14]

Immunosuppressive agents are the mainstay of therapy for diffuse alveolar hemorrhage, especially if associated with systemic or pulmonary vasculitis. In those with life-threatened vital organ loss, initial therapy requires high-dose cyclophosphamide and prednisolone for the induction of remission.[15] Besides corticosteroids, other immunosuppressive drugs such as cyclophosphamide, azathioprine, mycophenolate mofetil, methotrexate, and etanercept may be used in diffuse alveolar hemorrhage, especially when the condition is severe, when first-line therapy with corticosteroids has proven ineffective or when a specific underlying cause is present.[16] Azathioprine is also recommended for maintenance therapy. A recent multicenter study suggested that changing 3-month cyclophosphamide to azathioprine to maintain remission is as effective as a more prolonged course of cyclophosphamide.[17] Relapse is common despite continued immunosuppressive therapy, with 50% of patients relapsing within 5 years. Besides these, trials are on to see the role of newer drugs like rituximab, deoxyspergualin, antithymocyte globulin, and interferons in WG.[18]

In conclusion, we can say that this complication has a high mortality rate and may precede other evidence of disease. Early diagnosis is imperative, since aggressive therapy with high-dose corticosteroids, cytotoxic agents, and sometimes plasmapheresis may be beneficial. Radiologists should be aware of the possibility of pulmonary hemorrhage when confronted with diffuse pulmonary parenchymal abnormalities, especially if there is clinical evidence of unexplained blood loss, or signs and symptoms of collagen vascular disease.
